# HIV-tuberculosis co-infection in Liangshan, a resource-limited region of Southwest China: trends and implications in the context of integrated prevention, 2019–2024

**DOI:** 10.1186/s40249-026-01495-w

**Published:** 2026-09-04

**Authors:** Xuefei Bai, Ju Wang, Gang Yu, Jinhong Shi, Sisi Fan, Ruobing Wang, Yubing Wang, Xiaoying Feng, Rong Pei, Rujun Liao

**Affiliations:** 1 Sichuan Center for Disease Control and Prevention, Chengdu, 610041 Sichuan China; 2 Liangshan Center for Disease Control and Prevention, Liangshan Yi Autonomous Prefecture, Xichang, 615000 Sichuan China; 3 School of Public Health, Chengdu University of Traditional Chinese Medicine, Chengdu, 611137 Sichuan China

**Keywords:** HIV, Tuberculosis, Co-infection, Integrated prevention and control, Epidemiological trend, Drug resistance, Liangshan Yi Autonomous Prefecture, Resource-limited region, Yi “clan”(*Jiazhi*) system, China

## Abstract

**Background:**

Tuberculosis (TB) and HIV co-infection remains a major public health challenge in resource-limited settings. Liangshan Yi Autonomous Prefecture in Southwest China bears a dual high burden of HIV and TB. This study aimed to analyze the epidemiological trends of HIV-TB co-infection in Liangshan from 2019 to 2024 and to inform integrated prevention strategies.

**Methods:**

A retrospective analysis was conducted on 3367 HIV-TB co-infected cases extracted from the National HIV/AIDS and TB surveillance systems. Records were linked via unique identifiers. Descriptive statistics and chi-square tests for trend assessed incidence trends, demographics, transmission routes, treatment outcomes, and drug resistance.

**Results:**

From 2019 to 2024, HIV-TB incidence declined from 12.26 to 8.76 per 100,000 (*χ*^2^_trend_ = 40.459, *P* < 0.001), driven by high-burden counties and populations under 45 years (all *P* < 0.001). Patients were predominantly young males (61.10%) and farmers (92.37%). The main HIV transmission routes were heterosexual contact (48.29%) and injection drug use (41.31%), with marked sex and age disparities: injection drug use predominated in males (50.92%) and those aged 30 – < 45 years (50.32%), while heterosexual transmission prevailed in females (77.07%) and older adults (≥ 60 years, 87.62%). Treatment success rate was 81.76%, significantly lower in the elderly (69.52%, *P* < 0.05). Any drug resistance (3.30%) increased from 1.54% to 3.10% (*χ*^2^_trend_ = 8.481,* P *_trend_ = 0.004).

**Conclusion:**

During the implementation of integrated strategies, HIV-TB incidence showed a declining trend, particularly in high-burden areas and younger populations. Persistent challenges—stagnant incidence and poor outcomes in the elderly, high loss to follow-up (10.87%), rising drug resistance—require targeted interventions: leveraging the Yi “clan” (*Jiazhi*) system, strengthening active screening and nutritional support for older adults, optimizing drug resistance surveillance, and integrating digital health tools.

**Graphical Abstract:**

**Figure:**
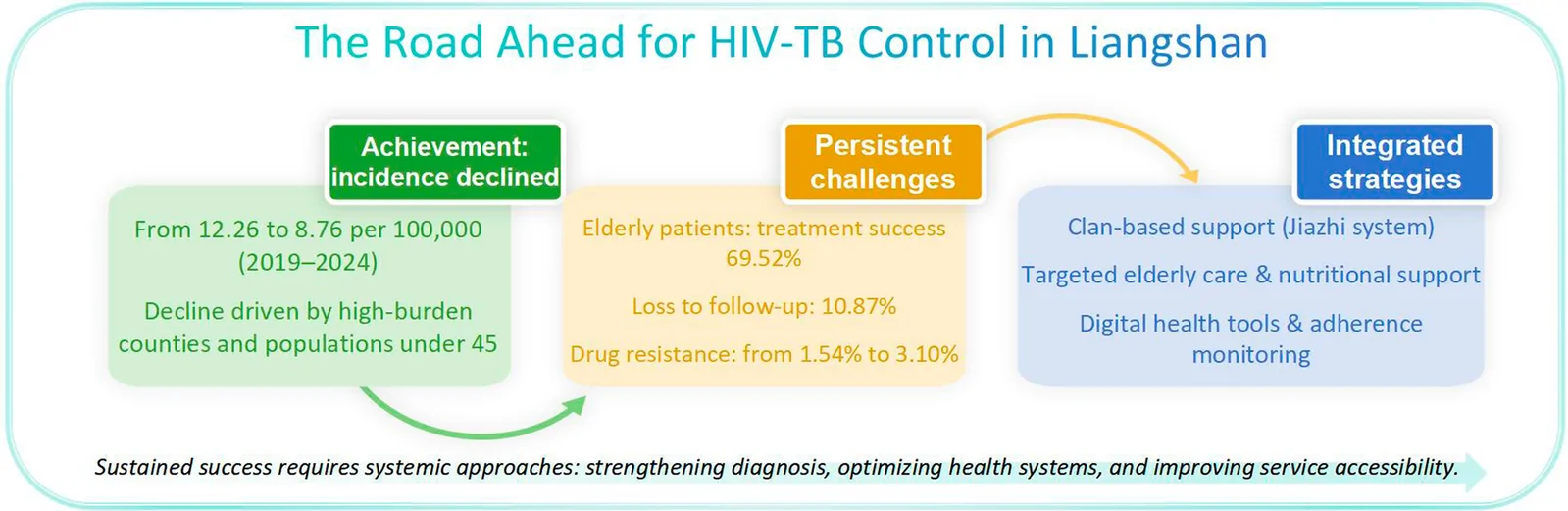

## Background

Tuberculosis (TB) remains a serious global public health threat. According to the World Health Organization’s *Global Tuberculosis Report 2025*, an estimated 10.7 million people developed TB globally in 2024, with an incidence rate of 131 per 100,000 population. From 2015 to 2024, the global incidence fell by only 12.3%—far short of the End TB Strategy’s 2025 target of a 50% reduction [1, 2]. In China, an estimated 696,000 new TB cases occurred in 2024 (incidence: 49 per 100,000, down 5.8% from 2023). China has dropped from third to fourth globally and, for the first time, entered the medium–low burden category [1]. However, this rate remains well above the 2035 target of 10 per 100,000, underscoring the continued urgency of targeted TB control in high-risk regions.

Within China, Sichuan Province is a key region for persistent TB transmission, with marked spatial heterogeneity in its epidemic distribution [3]. Liangshan Yi Autonomous Prefecture bears one of the heaviest TB burdens in Sichuan. During the 13th Five-Year Plan period (2016–2020), the reported pulmonary TB incidence in Liangshan consistently ranked among the highest in the province. This situation is closely linked to socioeconomic vulnerabilities, including geographic remoteness, economic underdevelopment, and limited healthcare resources [4, 5]. Key populations, including ethnic minorities and children in rural areas—face a disproportionately high TB incidence [6]. This burden is compounded by significantly low vaccination coverage among ethnic minority children, which poses an additional transmission risk [7].

In addition to its high TB burden, Liangshan also faces a severe and long-standing HIV epidemic. As the largest Yi-inhabited area in China and a resource-limited region, Liangshan has a distinct socio-cultural context that shapes its HIV epidemic landscape. Local studies have reported demographic and behavioral factors associated with HIV transmission in this population [8, 9].In recent years, the local HIV epidemic shifted from concentrated transmission among high-risk groups (e.g., people who inject drugs) to general population spread, with heterosexual transmission gradually replacing injection drug use as the main route, reflecting the increasing complexity of the local transmission network [10, 11]. This community-based, widespread HIV transmission pattern creates conditions conducive to the synergistic spread of opportunistic infections such as TB.

The convergence of these two epidemics presents a major global public health challenge. They exacerbate each other pathologically: HIV infection not only increases the risk of latent TB reactivation and progression to active disease but also complicates TB diagnosis and treatment, leading to significantly higher mortality among co-infected patients [12, 13]. Globally, TB is the leading cause of death among people living with HIV, with case fatality rates far exceeding those in HIV-negative individuals [1]. Global Burden of Disease studies show that the burden of HIV-TB co-infection falls most heavily on young adults and is strongly negatively correlated with the Socio-demographic Index (SDI) [14]. Low- and middle-income countries with a high disease burden face escalating challenges in clinical diagnosis and treatment delivery [15–19].

These challenges are equally evident in China, where TB is one of the most common opportunistic infections among people living with HIV [20]. Reports from several provincial-level administrative divisions (PLADs)—including Guangxi and Guizhou—indicate that HIV-TB co-infection has emerged as a critical public health issue affecting local populations [21–24]. This dual epidemic is particularly prominent in Liangshan. As early as 2008, Butuo County became the first county in China where the proportion of reported people living with HIV/AIDS exceeded 1% of the permanent population [25]. Local studies have found an HIV co-infection rate of 14.4% among hospitalized smear-positive pulmonary TB patients [26]. The two diseases exhibit significant spatial and demographic overlap, with synergistic transmission effects, posing a persistent threat to high-risk groups such as children and ethnic minorities [27–29].

In response to the severe HIV-TB co-infection crisis in Liangshan, the Chinese government implemented two core interventions: the Integrated Prevention and Control of Four Diseases (IPC4D) policy and the grassroots 1 + M + N + P precision prevention and control model.

The IPC4D policy was formally implemented in 2021, integrating HIV, tuberculosis, hepatitis C, and syphilis into a unified comprehensive prevention and control framework. Leveraging the overlapping transmission routes of HIV, sexually transmitted infections, and hepatitis C, as well as the high co-infection rates of HIV with tuberculosis, hepatitis C, and syphilis, the policy integrated screening, diagnosis, treatment, and follow-up services. In accordance with the principle of opt-out consent, regular testing services were provided through medical institutions at all levels to expand screening coverage and standardize case management, aiming to increase early diagnosis and treatment rates, reduce new infection and transmission risks, and alleviate the regional disease burden.

The 1 + M + N + P model was first piloted as “1 + M + N” in four high-burden counties (Butuo, Zhaojue, Yuexi, and Meigu) in 2017, it was upgraded to “1 + M + N + P” in 2021 and expanded to all 17 counties and cities in Liangshan Prefecture. In this model: 1 refers to the township government (responsible for resource coordination and prioritizing mobile people living with HIV and treatment-failure patients), M to township medical staff (technical support and treatment management), N to village-level HIV prevention workers (home visits, directly observed medication guidance, and health education), and P to grassroots public security departments (participating in comprehensive social governance and conducting drug control and HIV prevention efforts). It strengthens grassroots grid-based precision management and improves service accessibility in remote areas.

The two measures complement each other: IPC4D unifies multi-disease prevention and control standards at the population level, while 1 + M + N + P ensures precise grassroots implementation, forming a replicable model for comprehensive prevention and control in high-burden regions.

Attributable to targeted policy support and increased financial investment, HIV and TB screening efforts in Liangshan have been systematically expanded, and health education activities have been innovatively promoted to raise public awareness and reduce disease-related discrimination [30]. However, local prevention and control efforts still face considerable challenges: uneven resource allocation, with TB (particularly drug-resistant TB) remaining chronically underfunded; health workforce shortages, excessive workload at the grassroots level, and widespread professional burnout [31]; and timely treatment initiation and patient adherence continue to be affected by disease-related stigma, economic burden, and cultural and language barriers—all of which increase the risk of adverse outcomes for anti-tuberculosis treatment (ATT) [32].

In this context, analyzing the epidemiological trends of HIV-TB co-infection in Liangshan based on surveillance data serves two key purposes: to evaluate the interim effectiveness of the IPC4D policy and to identify critical weak links in real-world prevention and control practices. By objectively characterizing population-level trends, this analysis can help consolidate and optimize local control strategies and provide important insights for other resource-limited regions with a high burden of HIV-TB co-infection.

## Methods

### Study design and setting

We conducted a retrospective case study in Liangshan Yi Autonomous Prefecture, Sichuan Province, a resource-limited region in Southwest China with a population of approximately 5.5 million and a high dual burden of HIV and tuberculosis. Liangshan lies in mountainous southwestern Sichuan, and features a subtropical monsoon climate and varied topography; its remote geography and underdeveloped transportation infrastructure have long posed challenges to healthcare access. During the 13th Five-Year Plan period (2016–2020), the prefecture consistently reported one of the highest TB burdens in the province [4, 5], while the HIV epidemic has also been severe, with Butuo County becoming, as early as 2008, the first county in China in which reported HIV/AIDS prevalence exceeded 1% of the permanent population [25].

### Data sources and linkage

In China, both HIV and TB are statutorily notifiable diseases, and confirmed cases must be reported to the national surveillance system within 24 h. Data on HIV and TB cases among permanent residents of Liangshan Prefecture from 2019 to 2024 were obtained from the Chinese HIV/AIDS Comprehensive Response Information Management System (CRIMS) and the Tuberculosis Information Management System (TBIMS) of the Chinese Center for Disease Control and Prevention. We extracted the TB database from the TBIMS and, in parallel, exported the HIV database (including both the real-time database and historical records from the CRIMS). All data were retrieved by designated personnel and encrypted prior to analysis.

**Fig. 1:**
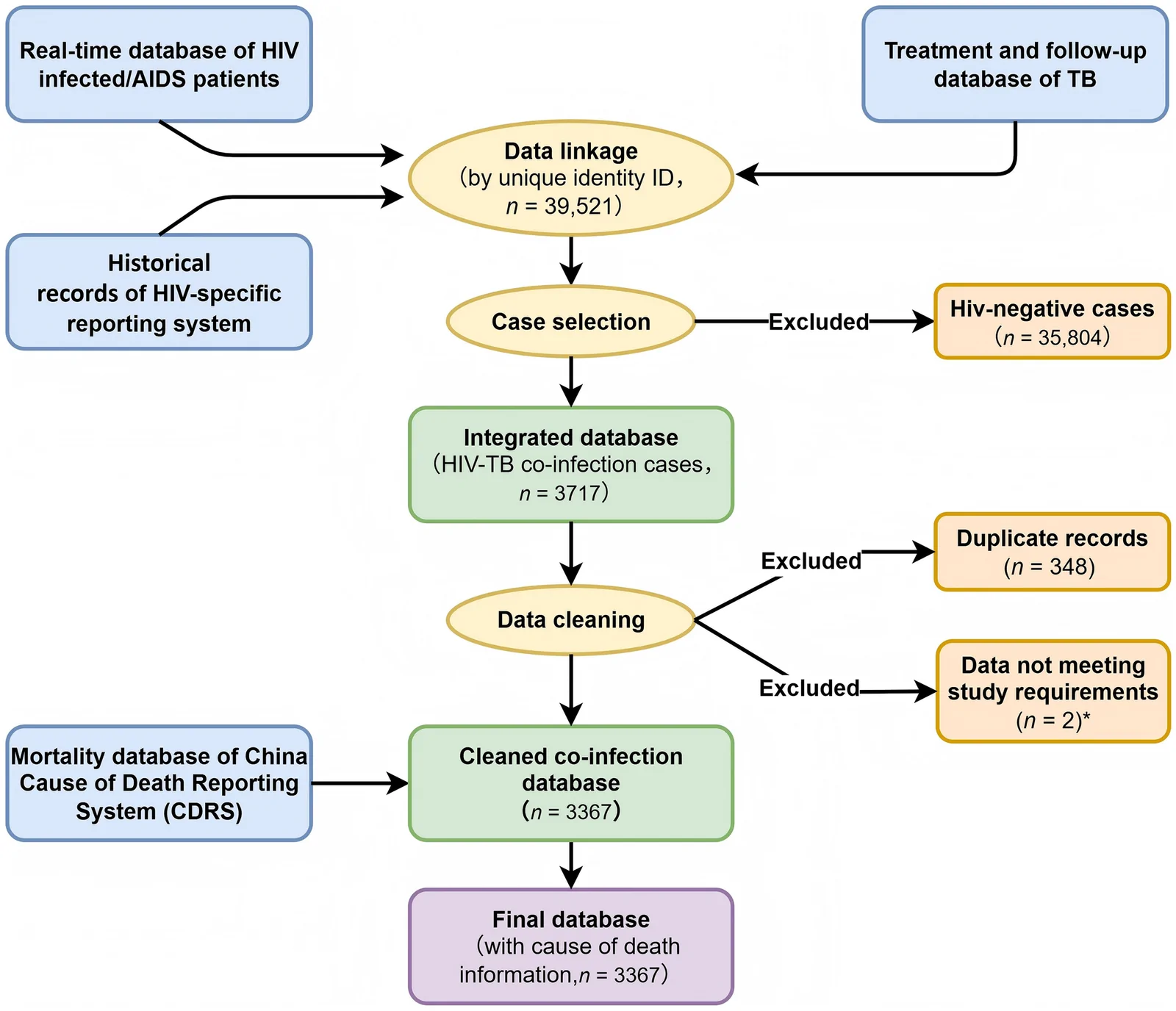
Data linkage and case selection flowchart for HIV-TB co-infection study, Liangshan Prefecture, 2019–2024. *These two cases were excluded because their diagnosis date was after December 31, 2024

Individual records from the two databases were linked using unique national identification numbers to create an integrated, de-identified database of HIV-TB co-infected cases. Demographic data and stratified population data (by sex and age group) for Liangshan Prefecture for the period 2019–2024 were obtained from the annual household registration records of the Sichuan Provincial Department of Public Security. Raw population data are not publicly available, so specific denominator values are not reported. For patients with recorded deaths during follow-up, causes of death were supplemented through linkage with the China Cause of Death Reporting System (CDRS) (Fig. 1).

### Study population and definitions

#### Data cleaning, study population and definitions

We analyzed records of incident TB cases among permanent residents in Liangshan Prefecture from 2019 to 2024. Incident HIV-TB co-infected cases were defined as newly notified tuberculosis cases recorded in the TBIMS, including recurrent tuberculosis after standard anti-tuberculosis treatment, and confirmed HIV-positive via unique national identification numbers matched in the CRIMS. Records were excluded if they had revised diagnoses, missing key treatment information, TB infection alone (without HIV co-infection), or duplicate entries (identified by unique ID and diagnosis date). Ultimately, 3367 patients with HIV-TB co-infection were included in the analysis. Definitions of HIV/AIDS, TB cases, and treatment outcomes in this study strictly adhered to China's current national health industry standards and technical specifications [33, 34], and were aligned with relevant World Health Organization (WHO) guidelines [35, 36]. All case information was reported in a standardized manner.

## Data analysis

We used SPSS version 30.0 (IBM Corp., Armonk, NY, USA) and Microsoft Excel 2007 (Microsoft Corp., Redmond, WA, USA) for statistical analyses. Descriptive statistics were performed on annual reported incidence, age, sex, ethnicity, marital status, education level, occupation, probable transmission routes, and anti-TB treatment outcomes among the included co-infected patients. Non-normally distributed continuous data are presented as median with interquartile range (P25, P75). The annual new infection rate was calculated as the number of incident cases divided by the average stratified household registration population for the corresponding year. The drug resistance rate was defined as the proportion of TB-resistant cases among newly reported HIV-TB co-infected cases in the corresponding period. The chi-square test was used for between-group proportion comparisons, and the chi-square test for trend was applied to assess temporal changes. Statistical significance was set at *α* = 0.05.

## Results

### Epidemiological trends and population profile

From 2019 to 2024, a total of 3367 HIV-TB co-infected cases were identified in Liangshan Prefecture, Sichuan Province. The majority of patients were male: 2547 cases (75.60%) were male and 820 (24.40%) were female. The median age was 37 years [interquartile range (IQR): 32–43]. The predominant age group was 30 – < 45 years, accounting for 61.10%, followed by 45 – < 60 years (18.40%), 15 – < 30 years (13.60%), < 15 years (3.70%), and ≥ 60 years (3.10%). Most patients were farmers (92.40%), with smaller proportions being students (2.90%), homemakers or unemployed individuals (1.10%), and other occupations.

The reported incidence of HIV-TB co-infection in Liangshan Prefecture showed a significant downward trend from 2019 to 2024 (*χ*^2^_*trend*_ = 40.459, *P*_*trend*_ < 0.001). According to local demographic data, incidence varied significantly across population subgroups over the six years: it was markedly higher in males (15.26 per 100,000) than in females (5.19 per 100,000) (*χ*^2^ = 793.847, *P* < 0.001). Differences in incidence by age group and county were also statistically significant (both *P* < 0.001). The highest incidence was in the 30 – < 45-year age group (33.08 per 100,000), followed by the 45 – < 60-year group (9.88 per 100,000). Butuo County (50.90 per 100,000), Zhaojue County (36.40 per 100,000), Meigu County (17.78 per 100,000), Yuexi County (17.41 per 100,000), and Jinyang County (17.24 per 100,000) ranked as the top five high-burden areas by incidence (Table 1).

**Table 1:** Reported incidence of HIV-TB co-infection in Liangshan Prefecture, 2019–2024, by demographic and geographic characteristics (*n* = 3367)

Characteristic	Reported cases,* n*	Reported incidence (per 100,000)	*χ*^2^/*χ*^2^_*trend*_	*P* value/*P*_*trend*_ value
Year of report				
2019	651	12.26	40.459*	< 0.001
2020	605	11.35		
2021	570	10.57		
2022	524	9.65		
2023	533	9.72		
2024	484	8.76		
Sex				
male	2547	15.26	793.847	< 0.001
female	820	5.19		
Age group, years				
< 15	126	1.51	4097.936	< 0.001
15 – < 30	459	5.97		
30 – < 45	2057	33.08		
45 – < 60	620	9.88		
≥ 60	105	2.67		
Place of residence				
Butuo County	675	50.90	4927.384	< 0.001
Zhaojue County	742	36.40		
Meigu County	307	17.78		
Yuexi County	401	17.41		
Jinyang County	225	17.24		
Puge County	202	15.10		
Xide County	163	12.28		
Ganluo County	149	10.26		
Xichang City	190	4.25		
Leibo County	66	3.79		
Mianning county	83	3.39		
Dechang County	42	3.18		
Yanyuan County	65	2.79		
Ningnan County	26	2.14		
Muli Tibetan Autonomous County	9	1.09		
Huidong County	12	0.47		
Huili City	10	0.36		

*Indicates chi-square test for trend

### Temporal trends and drivers of decline

Analysis of annual incidence trends among HIV-TB co-infected patients with different characteristics in Liangshan from 2019 to 2024 showed that the overall decline was primarily driven by geographic and age factors.

Geographically, the overall decline was mainly attributable to significant improvements in high-burden counties: Butuo, Zhaojue, Yuexi, Jinyang, and Xide—counties with higher baseline incidence—all showed significant downward trends in average annual reported incidence (all *P*_*trend*_ < 0.05), whereas counties with lower baseline incidence showed no temporal changes. By age, all age groups under 45 years showed significant declines (all *P*_*trend*_ < 0.001). For the 45 – < 60-year group, the downward trend approached the threshold of statistical significance (*χ*^2^_*trend*_ = 4.021, *P*_*trend*_ = 0.045), while the ≥ 60-year group showed no significant decline (*χ*^2^_*trend*_ = 2.757, *P*_*trend*_ = 0.097). The average annual incidence for both sexes showed downward trends over the six years (both *P*_*trend*_ < 0.001) (Table 2).

**Table 2:** Annual reported incidence of HIV-TB co-infection in Liangshan Prefecture, 2019–2024 (*n* = 3367)

Characteristic	2019		2020		2021		2022		2023		2024		*χ*^2^_*trend*_	*P*_*trend*_ value
	Cases, *n*	Incidence (per 100,000)	Cases, *n*	Incidence (per 100,000)	Cases, *n*	Incidence (per 100,000)	Cases, *n*	Incidence (per 100,000)	Cases, *n*	Incidence (per 100,000)	Cases, *n*	Incidence (per 100,000)		
Place of residence														
Butuo County	156	72.80	139	64.74	71	32.57	104	46.72	94	41.38	111	48.38	18.985	< 0.001
Zhaojue County	88	25.84	110	33.49	162	48.25	118	34.66	142	41.15	122	35.01	4.043	0.044
Jinyang County	64	30.04	36	16.78	45	20.78	26	11.91	32	14.52	22	9.91	24.217	< 0.001
Yuexi County	86	22.99	81	21.60	64	16.78	65	16.84	54	13.80	51	12.89	17.122	< 0.001
Xide County	47	20.85	21	9.72	29	13.22	24	10.86	22	9.89	20	8.95	10.005	0.002
Meigu County	50	17.95	49	17.47	44	15.40	56	19.30	60	20.38	48	16.16	0.030	0.863
Puge County	33	15.12	40	18.26	39	17.54	38	17.00	22	9.74	30	13.10	2.803	0.094
Ganluo County	33	13.97	19	7.99	31	12.83	22	9.04	28	11.39	16	6.46	3.293	0.070
Leibo County	21	7.38	19	6.65	9	3.11	4	1.37	6	2.04	7	2.36	17.879	< 0.001
Mianning County	20	4.94	23	5.68	12	2.94	7	1.72	12	2.91	9	2.18	9.317	0.002
Ningnan County	7	3.49	3	1.49	5	2.46	2	0.99	7	3.44	2	0.98	0.895	0.344
Yanyuan County	13	3.38	16	4.15	10	2.57	10	2.57	9	2.31	7	1.80	3.572	0.059
Xichang City	21	3.04	42	5.79	34	4.59	33	4.39	34	4.41	26	3.30	0.328	0.567
Muli Tibetan Autonomous County	4	2.89	1	0.73	0	0.00	1	0.73	2	1.45	1	0.73	1.145	0.285
Dechang County	5	2.29	2	0.92	11	4.99	11	4.98	5	2.25	8	3.60	1.069	0.301
Huidong County	3	0.70	2	0.47	1	0.24	2	0.47	1	0.24	3	0.71	0.024	0.878
Huili City	0	0.00	2	0.43	3	0.65	1	0.22	3	0.65	1	0.22	0.308	0.579
Age group, years														
< 15	30	2.12	34	2.40	15	1.07	20	1.44	16	1.16	11	0.81	12.905	< 0.001
15 – < 30	105	8.34	97	7.74	73	5.74	68	5.29	66	5.09	50	3.80	29.547	< 0.001
30 – < 45	405	39.41	367	35.86	352	34.53	324	31.49	331	31.54	278	26.02	31.095	< 0.001
45 – < 60	87	8.92	89	8.77	111	10.40	101	9.34	108	10.07	124	11.63	4.021	0.045
≥ 60	24	3.80	18	2.88	19	3.02	11	1.71	12	1.74	21	2.90	2.757	0.097
Sex														
Male	475	17.40	444	16.20	432	15.59	390	13.98	428	15.19	378	13.32	16.260	< 0.001
Female	176	6.82	161	6.21	138	5.26	134	5.07	105	3.94	106	3.94	33.145	< 0.001

### Characteristics of HIV transmission and TB treatment profiles

The HIV transmission routes, anti-TB treatment outcomes, and drug resistance profiles of newly reported HIV-TB co-infected patients in Liangshan from 2019 to 2024 are shown in Table 3. Heterosexual transmission was the predominant HIV route (48.29%), followed by injection drug use (41.31%) and mother-to-child transmission (3.42%); homosexual transmission accounted for the smallest proportion (0.30%).

**Table 3:** Characteristics of HIV transmission routes, anti-TB treatment outcomes, and drug resistance among HIV-TB co-infected patients in Liangshan Prefecture, 2019–2024 (*n* = 3367)

Characteristic	Cases, *n*	Proportion (%)
HIV transmission route		
Heterosexual transmission	1626	48.29
Injection drug use	1391	41.31
Sexual contact + injection drug use	137	4.07
Mother-to-child transmission	115	3.42
Unknown	63	1.87
Homosexual transmission	10	0.30
Other	25	0.74
Anti-TB drug resistance		
No drug resistance	3256	96.70
Rifampicin-resistant	52	1.54
Isoniazid-resistant	32	0.95
Multidrug-resistant (MDR)	26	0.77
Pre-extensively drug-resistant	1	0.03
Anti-TB treatment outcome		
Completed treatment or cured	2753	81.76
Transferred to MDR-TB treatment	65	1.93
Adverse TB treatment outcome	27	0.80
Death not due to TB	156	4.63
Lost to follow-up or unknown	366	10.87

*MDR-TB* multidrug-resistant tuberculosis

Regarding anti-TB treatment outcomes, 81.76% of patients completed treatment or were cured, approximately 1.93% were transferred to multidrug-resistant TB (MDR-TB) treatment, 0.80% experienced adverse treatment outcomes (adverse reactions, treatment failure, or TB-related death), and 10.87% were lost to follow-up or had unknown outcomes. Drug resistance testing revealed that 3.30% of co-infected patients had TB drug resistance. Among resistant cases, the main types were resistance to rifampicin (46.85%) and isoniazid (28.83%), followed by MDR-TB (23.42%).

### Stratified distribution of HIV transmission routes, drug resistance, and treatment outcomes

To further explore the distribution of the above characteristics across different populations, we performed a stratified analysis by sex and age group for HIV transmission routes, drug resistance, and anti-TB treatment outcomes (Table 4). By sex, there were statistically significant differences in HIV transmission routes and anti-TB drug resistance (both *P* < 0.05), while the difference in anti-TB treatment outcomes was not significant (*χ*^2^ = 5.446, *P* = 0.245). By age group, there were statistically significant differences in HIV transmission routes and anti-TB treatment outcomes (both *P* < 0.05), while the difference in anti-TB drug resistance was not significant (*χ*^2^ = 2.824, *P* = 0.588).

**Table 4:** Differences in clinical characteristic composition among HIV-TB co-infected patients stratified by gender and age (*n* = 3367)

Characteristic	Sex				Age group, years						
	Male	Female	*χ*^2^	*P* value	< 15	15—< 30	30—< 45	45—< 60	≥ 60	*χ*^2^	*P* value
HIV transmission route			501.741	< 0.001						2446.684	< 0.001
Heterosexual transmission	994 (39.03)	632 (77.07)			2 (1.59)	292 (63.62)	914 (44.43)	326 (52.58)	92 (87.62)		
Injection drug use	1297 (50.92)	94 (11.46)			1 (0.79)	98 (21.35)	1035 (50.32)	245 (39.52)	12 (11.43)		
Sexual contact + injection drug use	132 (5.18)	5 (0.61)			0(0.00)	11 (2.40)	85 (4.13)	41 (6.61)	0(0.00)		
Mother-to-child transmission	58 (2.28)	57 (6.95)			90 (71.43)	25 (5.45)	0(0.00)	0(0.00)	0(0.00)		
Unknown	39 (1.53)	24 (2.93)			25 (19.84)	17 (3.70)	14 (0.68)	6 (0.97)	1 (0.95)		
Homosexual transmission	10 (0.39)	0(0.00)			0(0.00)	4 (0.87)	5 (0.24)	1 (0.16)	0(0.00)		
Other	17 (0.67)	8 (0.98)			8 (6.35)	12 (2.61)	4 (0.19)	1 (0.16)	0(0.00)		
Anti-TB drug resistance			4.126	0.042						2.824	0.588
No drug resistance	2454 (96.35)	802 (97.80)			124 (98.41)	446 (97.17)	1990 (96.74)	596 (96.13)	100 (95.24)		
Rifampicin-resistant	42 (1.65)	10 (1.22)			1 (0.79)	5 (1.09)	36 (1.75)	7 (1.13)	3 (2.86)		
Isoniazid-resistant	27 (1.06)	5 (0.61)			1 (0.79)	6 (1.31)	17 (0.83)	7 (1.13)	1 (0.95)		
Multidrug-resistant (MDR)	23 (0.90)	3 (0.37)			0	2 (0.44)	13 (0.63)	10 (1.61)	1 (0.95)		
Pre-extensively drug-resistant	1 (0.04)	0(0.00)			0(0.00)	0(0.00)	1 (0.05)	0(0.00)	0(0.00)		
Anti-TB treatment outcome			5.446	0.245						28.749	0.026
Completed treatment or cured	2069 (81.23)	684 (83.41)			105 (83.33)	384 (83.66)	1698 (82.55)	493 (79.52)	73 (69.52)		
Transferred to MDR-TB treatment	54 (2.12)	11 (1.34)			1 (0.79)	8 (1.74)	40 (1.94)	13 (2.10)	3 (2.86)		
Adverse TB treatment outcome	18 (0.71)	9 (1.10)			1(0.80)	2(0.44)	22 (0.32)	2(5.00)	0(0.00)		
Death not due to TB	125 (4.91)	31 (3.78)			5 (3.97)	13 (2.83)	97 (4.72)	31 (5.32)	10 (9.52)		
Lost to follow-up or unknown	281 (11.03)	85 (10.37)			14 (11.11)	52 (11.33)	200 (9.72)	81 (13.06)	19 (18.10)		

Values in parentheses are column percentages. *MDR-TB* multidrug-resistant tuberculosis

These differences were primarily manifested in the fact that injection drug use was the predominant HIV route for males, while heterosexual transmission was predominant for females; the proportion of females who completed treatment or were cured was slightly higher than that of males. Regarding age groups, injection drug use was the main HIV route for those aged 30 – < 45 years (50.32%), whereas heterosexual transmission was the main route for all other age groups, notably reaching 87.62% in those aged ≥ 60 years. Importantly, the proportion of patients aged ≥ 60 years who completed treatment or were cured was less than 70% (69.52%), which was significantly lower than that of other age groups after Bonferroni correction (pairwise comparisons across five age groups with Bonferroni correction for 10 comparisons, *P* < 0.05).

### Drug resistance trends in the context of integrated prevention

From 2019 to 2024, the overall TB drug resistance rate among HIV-TB co-infected patients in Liangshan was 3.30%. Rifampicin resistance was the most common type (46.85% of resistant cases), followed by isoniazid resistance (28.83%) and MDR-TB (23.42%), while pre-extensively drug-resistant tuberculosis was rarely detected in this study population.

**Fig. 2:**
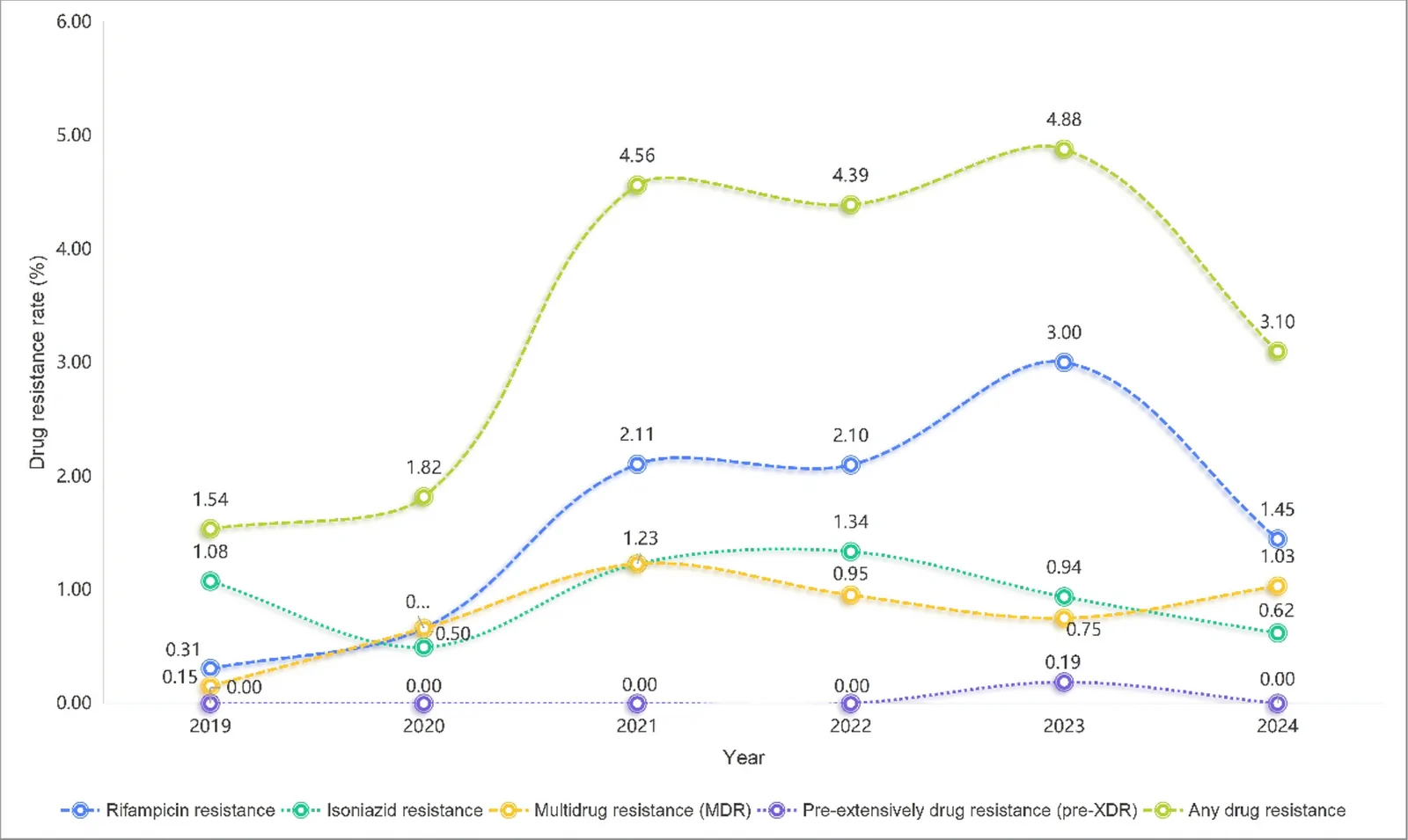
Trends in anti-TB drug resistance patterns among HIV-TB co-infected patients in Liangshan Prefecture, 2019–2024

Regarding temporal trends, though it slightly decreased in 2024, the overall drug resistance rate showed a significant upward trend (*χ*^2^_*trend*_ = 8.481, *P*_*trend*_ = 0.004), rising from 1.54% in 2019 to 3.10% in 2024, with the highest reported rate recorded in 2023 at 4.88%. Given the limited case numbers of individual drug-resistance subtypes, we did not perform separate trend tests for each category. Nevertheless, different drug-resistant subtypes showed distinct fluctuating characteristics during the six-year study period, which are visually presented in Fig. 2. The proportional composition of each resistance type fluctuated annually without stable regular variations.

## Discussion

Liangshan Prefecture in Sichuan Province is an area with a dual high burden of HIV and TB. HIV-TB co-infection in this region warrants particular attention and may provide important insights for other high-burden, resource-limited settings worldwide.

### Overall trends in the context of integrated prevention

During the implementation of the IPC4D policy and the “1 + M + N + P” precision prevention and control model, the reported incidence of HIV-TB co-infection in Liangshan showed a significant downward trend from 2019 to 2024 (from 12.26 per 100,000 to 8.76 per 100,000), consistent with reports from other regions in China [37]. However, global burden of disease models and local studies suggest that the age-standardized burden of HIV-TB in low-SDI regions may continue to rise in the coming years [1, 14]. Meanwhile, HIV incidence showed a decreasing trend in Liangshan during the study period [25], while TB incidence remained high with an upward risk, as documented in local surveillance and provincial projection models [38, 39]. These divergent trends may be related to local socioeconomic development and improvements in disease screening and reporting systems. Taken together, the observed decrease in co-infection rates cannot be attributed to a single intervention, and Liangshan's prevention and control achievements need sustained consolidation. Studies have indicated that relying solely on symptom-based screening is insufficient to detect all TB cases, recommending systematic TB screening for people living with HIV during every healthcare encounter to reduce diagnostic delays [40]. This evidence offers valuable insights for optimizing screening strategies in Liangshan.

### Geographic and demographic drivers of decline

The demographic profile of HIV-TB co-infected patients in Liangshan from 2019 to 2024 was highly concentrated, predominantly involving young adult males (30–45 years) and farmers (92.37%). This epidemiological feature differs from the age-related increase of tuberculosis in the general population; it is closely related to local HIV epidemic characteristics and is supported by evidence from other countries and Chinese PLADs [13, 23, 37, 41]. The decline in incidence in high-burden counties such as Butuo, Zhaojue, Yuexi, and Jinyang was a major driver of the Liangshan's overall decline. This reflects the effectiveness of the Chinese government's strategy of concentrated resource allocation and precision interventions (e.g., expanded screening, community mobilization, multi-sectoral collaboration) in key disease control areas. The significant decline in all age groups under 45 years suggests a good response to prevention and control measures among the younger population. The downward trend in incidence for both sexes indicates that local interventions have achieved good coverage across genders. These findings suggest that Liangshan's prevention and control strategies may have yielded initial progress in key areas and populations, which could provide useful insights for other high-burden regions.

### Persistent challenges: the neglected older adults

This study showed that the incidence rate did not exhibit a significant declining trend among individuals aged 60 years and above during the study period. This finding may be influenced by the small sample size of this subgroup, potentially resulting in limited statistical power. Given that the treatment success rate in this age group was 69.52%, markedly lower than that in other age groups, close attention should still be paid to the risk of co-infection and adverse treatment outcomes among the elderly. Previous studies have indicated that elderly patients commonly encounter various practical barriers to active screening and regular follow-up [42]. In addition, inadequate social support—such as empty-nest living, bereavement, and adult children working away from home—may lead to insufficient supervision and daily care during treatment, which could adversely affect clinical outcomes [43]. In light of these findings, we recommend strengthening sexual health education and disease prevention for this population, promoting active screening, implementing nutritional support programs, and simplifying treatment procedures.

### Transmission route syndemic and its implications

The HIV transmission routes among co-infected patients in this study reveal a deeper “syndemic” pattern: injection drug use predominates in males (50.92%), while heterosexual transmission predominates in females (77.07%); injection drug use is dominant in the 30 – < 45-year age group (50.32%), whereas heterosexual transmission is dominant in all other age groups, reaching 87.62% in those aged ≥ 60 years. This pattern reflects the complexity of HIV transmission within Liangshan's unique socio-cultural context. This syndemic presents multidimensional requirements for prevention and control. First, young adult male farmers face both drug-related risks and difficulties in regular follow-up due to high mobility, necessitating strengthened pre-migration screening and mobile population management [31]. Second, women infected through heterosexual transmission require support to enhance their negotiation skills in sexual activities and access to diverse protective measures [8]. Third, the varying transmission characteristics across age groups call for targeted sexual health education for those aged ≥ 60 years, and integrated interventions—combining drug prevention, sexual health promotion, and family-based approaches—for the “mixed” transmission pattern seen in the 30 – < 45-year age group [44, 45].

### Treatment outcomes, loss to follow-up, and the threat of drug resistance

The treatment success rate in this study was 81.76%, lower than the provincial average for Sichuan during the same period (89.01%) [4]. The proportion of patients lost to follow-up or with unknown outcomes was 10.87%, far exceeding that in the general TB population and consistent with findings from an international multi-center study [46]. Low treatment adherence and high loss to follow-up in this setting stem from multiple interacting factors. Previous studies have indicated that long TB treatment duration, medication burden, adverse drug reactions, and potential toxicities when combined with antiretroviral therapy (ART) may affect adherence [47]. These are further exacerbated by low health literacy, economic hardship, and lack of family support [48]. At the health system level, "N"-level personnel such as village doctors and HIV prevention officers, working under the integrated service model, face dramatically increased workloads, understaffing, heavy caseloads, and insufficient funding, leading to less intensive follow-up supervision [31]. These factors may compromise timely follow-up and intervention for patients with poor adherence, such as some older adults. Another local study found a paradoxically higher risk of death among patients followed up at county-level hospitals or above [49], suggesting that reliance solely on higher-level medical institutions is insufficient. Strengthening the tripartite "hospital-community-family" linkage model locally—by promoting consistent medication supervision and supportive care at the grassroots level, and developing objective adherence assessment methods—is needed to address previous shortcomings in long-term follow-up management [50].

Loss to follow-up not only potentially signifies treatment failure but also acts as a breeding ground for drug resistance. Studies indicate that Liangshan's high drug-resistant TB rate—among the highest in China—is primarily due to patient non-adherence rather than primary resistance to drug-resistant strains [31]. The observed upward trend in the overall drug resistance rate, may serve as a potential indicator of suboptimal treatment adherence among local co-infected patients. Therefore, it is crucial to strengthen drug resistance surveillance among high-risk groups (males, older adults, those with low education, unmarried/divorced individuals, and farmers), promote rapid molecular testing, explore digital health tools to support supervision [50–52], and consider providing tangible support such as transportation subsidies and nutritional packages. Simultaneously, alleviating the workload of grassroots "N"-level personnel is essential to ensure follow-up quality [30, 53]. Through these comprehensive measures, the emergence of additional drug-resistant TB patients can be curbed.

This study has several limitations. First, no local HIV and TB incidence data perfectly match the study period, so the exact driver of the declining co‑infection rate cannot be clearly identified. Second, this study was conducted based on routine disease control practice and population surveillance data, with no control group established; the time spans before and after policy implementation were unbalanced, and no appropriate time points were selected for comparative analysis. Therefore, the results may be affected by temporal confounding factors such as annual fluctuations and seasonal variations. Third, the population denominator data lacked income and occupational stratification, limiting stratified incidence analyses; occupation was recorded in the case data but was not fully integrated into multivariable analyses, and the surveillance data do not fully capture socioeconomic factors such as residents’ economic status. Fourth, several subgroups had relatively small sample sizes, which may reduce the statistical power of trend tests and affect the reliability of temporal trend analysis results. Finally, although official annual data on bidirectional screening coverage between HIV and TB patients are unavailable locally, such screening has been widely implemented in routine clinical and public health practice; therefore, the data used in this study can roughly reflect the actual co‑infection status in the study area. In light of the above considerations, this study, based on routine surveillance data, characterizes the epidemiological profile of HIV‑TB co‑infection and highlights key concerns. It explores the potential roles of relevant policies without establishing definite causal relationships. Further well‑designed controlled studies are warranted to validate these findings.

## Conclusions

Drawing on surveillance data of HIV-TB co-infection in Liangshan Prefecture from 2019 to 2024, this study provides a comprehensive analysis of epidemiological trends and infection characteristics within the context of integrated prevention. Key findings include a declining incidence trend, which occurred during the implementation of the ‘Four Diseases Co-prevention’ policy. The decline was primarily driven by high-burden counties and younger populations, suggesting a potential role of targeted precision interventions. The lack of improvement in incidence and low treatment success rate among older adults highlight a critical weak link in current control efforts, while loss to follow-up and emerging TB drug resistance threaten the progress achieved.

Building on these findings, we propose four priorities for comprehensive prevention and control in Liangshan and similar high-burden settings:

Place-based strategies: harnessing indigenous social resources. In high-burden counties such as Butuo and Zhaojue, the workload for grassroots follow-up is immense, with village doctors and HIV prevention officers facing staff shortages. Drawing on the Yi “clan” (*Jiazhi*) system, collaboration with clan leaders could be explored to integrate patient follow-up and medication supervision into traditional social networks, thereby improving intervention sustainability and cultural appropriateness.

People-centered approaches: implementing precision interventions. For older adults, we recommend establishing active screening mechanisms (e.g., integrated into local chronic disease management clinics), providing simplified treatment regimens, and strengthening nutritional support. For women, empowerment through women's health groups and peer education can enhance sexual health autonomy. For young adult male farmers, integrated screening should be delivered around key periods such as pre-migration and Spring Festival return, with improved follow-up for mobile populations.

Resource-optimized initiatives: filling gaps in drug resistance prevention and control. Given that rifampicin resistance accounts for a large proportion of overall drug resistance cases, and that local funding for TB (especially drug-resistant TB) remains substantially lower than for HIV, targeted interventions are urgently needed. We recommend expanding rapid drug resistance testing for all HIV-TB patients, establishing dedicated follow-up pathways for drug-resistant cases, and increasing financial support and performance incentives for key diagnostic procedures.

Technology- and support-integrated efforts: improving adherence management efficiency. Digital health tools (medication reminder apps, wearable devices) can be deployed to support supervision, with family engagement. Concurrently, tangible support such as transport subsidies and nutritional supplements can reduce patient burden, while task rationalization and performance incentives can alleviate burnout among frontline workers.

Global evidence indicates that HIV-TB co-infection control is closely tied to socioeconomic development. In low-SDI settings, disease control is particularly challenging, and expanding treatment coverage alone is insufficient to rapidly reduce disease burden [20]. The Liangshan experience suggests that policy integration and targeted resource investment may contribute to meaningful progress, but sustained success requires a systemic approach: strengthening grassroots diagnostic capacity, optimizing health system support, improving education for key populations, and improving service accessibility. Future research should prioritize tracing and characterizing patients lost to follow-up to generate further evidence for optimizing follow-up management and care retention.

## Data Availability

The data that support the findings of this study are available from the Liangshan Center for Disease Control and Prevention, but restrictions apply to the availability of these data, which were used under license for the current study and so are not publicly available. Data are, however, available from the corresponding authors upon reasonable request and with permission of the Liangshan Center for Disease Control and Prevention.

## Abbreviations

ART: Antiretroviral therapy
ATT: Anti-tuberculosis treatment
BMI: Body mass index
CDRS: China Cause of Death Reporting System
CRIMS: National Comprehensive HIV/AIDS Information Management System
HIV: Human Immunodeficiency Virus
IPC4D: Integrated prevention and control of four diseases
IQR: Interquartile range
MDR: Multidrug-resistant
MDR-TB: Multidrug-resistant tuberculosis
pre-XDR: Pre-extensively drug resistance
SDI: Socio-demographic Index
TB: Tuberculosis
TBIMS: Tuberculosis Information Management System
WHO: World Health Organization

## References

[1] World Health Organization. Global tuberculosis report 2025. Geneva: WHO; 2025.

[2] World Health Organization. End TB strategy. Geneva: WHO; 2019.

[3] Li T, Cheng Q, Li C, Stokes E, Collender P, Ohringer A, et al. Evidence for heterogeneity in China’s progress against pulmonary tuberculosis: uneven reductions in a major center of ongoing transmission, 2005–2017. BMC Infect Dis. 2005;19:615. 10.1186/s12879-019-4262-2 PMC6626433 31299911

[4] Lu J, Li T, Wang DX, Xia Y, Xiao Y, Chen C, et al. Epidemiological characteristics of tuberculosis in Sichuan during the 13th Five-Year Plan period. Dis Surveill. 2021;36(11):1147–51.

[5] Wei W, Xia L, Wu JL, Zhou ZL, Zhang WQ, Luan RS, et al. The environmental and socioeconomic effects and prediction of patients with tuberculosis in different age groups in Southwest China: a population-based study. JMIR Public Health Surveill. 2023;9:e40659. 36456535 10.2196/40659 PMC9883735

[6] Yu XR, Wang SJ, Yang XM, Fang M, Zeng X, Qi H, et al. Analysis of changes in reporting and diagnosis of pulmonary tuberculosis among children in Liangshan Yi Autonomous Prefecture, Sichuan Province from 2019 to 2021. Chin J Prev Med. 2023;57(8):1153–9. 10.3760/cma.j.cn112150-20230315-00192 37574305

[7] Liang L, Xiong QQ, Wei ZX, Xie FH, Li HM, Wu GH, et al. Characteristics and predictors of mortality among infants and toddlers hospitalized with tuberculosis: a ten-year case series study in Sichuan, China. BMC Infect Dis. 2025;25:785. 40457204 10.1186/s12879-024-10323-1 PMC12128501

[8] Zhang LL, Liu L, Lai WH, Feng L, Zhou JS. Comprehensive HIV/AIDS programs in Sichuan. In: HIV/AIDS in China: epidemiology, prevention and treatment. Singapore: Springer; 2019. p. 629–51.

[9] Yang SJ, Luo M, Zhang SH, Yao YN, Wang QX, Liao Q, et al. Overview on culture and customs related to AIDS epidemic and prevention among Yi people in Liangshan. Chin J AIDS STD. 2017;23(3):271–4.

[10] Yang H, Li YP, Xu MJ, Hu Y, Yuan FS, Liu LH, et al. The update of HIV-1 prevalence and incidence and spatio-temporal analyses of HIV recent infection among four sub-groups in Sichuan, China during surveillance period between 2016 and 2022. Infect Drug Resist. 2023;16:6535–48. 37814665 10.2147/IDR.S428744 PMC10560476

[11] Meng JY, Chen WZ, Zeng YL. Investigation of decreasing gender ratio of reported HIV/AIDS cases in Liangshan Yi Autonomous Prefecture, Sichuan Province: a mixed methods study [dissertation]. Chengdu: Chengdu Medical College; 2025

[12] Li YW, Li X. Epidemic status and research progress of AIDS/tuberculosis double infection. Chin Foreign Med Res. 2022;20(16):181–4.

[13] Wu HX, Zhu LY, Bao RA, Luo SY, Peng L, Huang X, et al. Global prevalence of HIV and *Mycobacterium tuberculosis* co-infection: a systematic review and meta-analysis of 371 included articles. Public Health. 2025;249:106034. 41205521 10.1016/j.puhe.2025.106034

[14] Tian XB, Wang C, Hao ZH, Chen JJ, Wu NP. Global, regional, and national burden of HIV and tuberculosis and predictions by Bayesian age-period-cohort analysis: a systematic analysis for the Global Burden of Disease Study 2021. Front Reprod Health. 2024;6:1475498. 39720120 10.3389/frph.2024.1475498 PMC11666487

[15] Zhang SX, Wang JC, Yang J, Lv S, Duan L, Lu Y, et al. Epidemiological features and temporal trends of the co-infection between HIV and tuberculosis, 1990–2021: findings from the Global Burden of Disease Study 2021. Infect Dis Poverty. 2024;13:59. 39152514 10.1186/s40249-024-01230-3 PMC11328430

[16] Preethi DP, Vanisree D, Parameswari K, Preethi H, Neeraja P. Determination of HIV status in Truenat positive tuberculosis patients: an observational study. Clin Microbiol. 2024;14(6):37–8.

[17] Rabelo LDBC, Oliveira K, Sobral R, Maquiné JEC, Dutra ASS, Souza LCOA. Estudo epidemiológico dos casos de coinfecção HIV/Tuberculose no Brasil entre os anos de 2019–2023. Braz J Implantol Health Sci. 2024;6(2):2155–66.

[18] Huang A, Chen HJ, Li JL, He YY, Chen P, Yang J, et al. Prevalence and treatment of patients with TB and HIV co-infection in Guizhou province, 2011–2020. Chin J Public Health. 2023;39(5):633–8.

[19] Chen C, Fu H, Liu YL, Chen SQ, Wang F, Liu RR, et al. Analysis on disease burden and epidemiological trends of HIV/AIDS co-infection with drug-resistant tuberculosis in China on the global perspective. Chin J Dis Control Prev. 2025;29(8):949–55.

[20] Li L, McGoogan JM, Wu Z. Common HIV co-infections in China: HBV, HCV, and TB. In: HIV/AIDS in China: epidemiology, prevention and treatment. Singapore: Springer; 2019. p. 63–73.

[21] Jiang X, Bai YL, Ma JJ, et al. Screening and treatment of TB/HIV co-infection in Jilin Province from 2016 to 2022. Chin J Public Health Eng. 2024;23(5):598–601.

[22] Abuduwaili K, Aihaiti Y, Wang MZ, Wang XQ. Analysis of epidemiological features of TB/HIV co-infection in Xinjiang from 2011 to 2020. Bull Dis Control Prev. 2025;40(1):34–8.

[23] Zhang X, Lang SL, Xu LJ, Gao YL. Analysis of epidemic characteristics and treatment of HIV-TB co-infection in Inner Mongolia Autonomous Region, 2016–2023. Chin J Antituberc. 2024;46(Suppl 1):50–5.

[24] Zhang P, Zhao AH, Gao M, Zhen XA, Yao YX, Jiang JG, et al. Analysis of epidemiological characteristics of TB/HIV co-infection in Henan Province from 2016 to 2021. In: Proceedings of the 34th National Academic Conference of the Chinese Antituberculosis Association; 2023. (in Chinese)

[25] Liu ZF, Tang XF, Liu YF, Zhang LL, Yang YH, Zheng YL, et al. HIV prevention and health poverty alleviation — Liangshan Prefecture, Sichuan Province, China, 2017–2020. China CDC Wkly. 2017;3(48):1031–5. 10.46234/ccdcw2021.250 PMC8633550 34888120

[26] Wang T, Zhou CX, Shang L, Zhou XY. Comorbidity and drug resistance of smear-positive pulmonary tuberculosis patients in the Yi autonomous prefecture of China: a cross-sectional study. BMC Infect Dis. 2023;23:586. 37674123 10.1186/s12879-023-08568-3 PMC10483793

[27] Wang DM, Wang C, An Q, Yan Q, Liao Y. Clinical characteristic, common sites, and geographical distribution of pediatric tuberculosis patients in Southwest China. Front Public Health. 2024;12:1234567. 10.3389/fped.2024.1327648 PMC10982491 38562135

[28] Wang DM, An Q, Yang Q, Liao Y. Epidemiology of drug-resistant tuberculosis among hospitalized children with tuberculosis in southwest China, 2017–2024. Front Microbiol. 2017;16:1609146. 10.3389/fmicb.2025.1609146 PMC12263897 40673150

[29] Wang DM, An Q, Yang Q, Liao Y. Prevalence and related factors of TB/HIV co-infection among hospitalized children with tuberculosis in Southwest China. Front Cell Infect Microbiol. 2025;15:1571291. 40568707 10.3389/fcimb.2025.1571291 PMC12187718

[30] Li J, He JG, Li T, Li YK, Gao WF. Analysis of feasibility and effectiveness of the implementation of strengthened tuberculosis management model in Liangshan Yi Autonomous Prefecture. Sichuan Province J Tuberc Lung Dis. 2022;3(6):540–5.

[31] Bi RL, Pei R, Jike CN, Yu G, Wang J, Wang ZH. Healthcare workers’ experiences with integrated HIV and TB prevention in Liangshan, China: a qualitative exploration of barriers and enablers. Front Public Health. 2025;13:1615781. 41164833 10.3389/fpubh.2025.1615781 PMC12558838

[32] Liao RJ, Hu L, Yu J, Chen Y, Chen MS, Yan JM. Association between TB delay and TB treatment outcomes in HIV-TB co-infected patients: a study based on the multilevel propensity score method. BMC Infect Dis. 2024;24(1):457. 38689228 10.1186/s12879-024-09328-7 PMC11061920

[33] National Health Commission of the People's Republic of China. Diagnosis for AIDS and HIV infection: WS 293–2019. 2019. http://www.nhc.gov.cn/wjw/s9491/201901/a8c9a79c2e134c81b6ddfd5c245fc232.shtml. Accessed 28 Feb 2026

[34] Chinese Center for Disease Control and Prevention. Technical guidelines for tuberculosis prevention and control in China. 2022. http://www.chinacdc.cn/jkzt/crb/zl/jhb/jsbw/202204/P020220414343625456660.pdf. Accessed 30 June 2026

[35] World Health Organization. Definitions and reporting framework for tuberculosis—2013 revision: updated December 2014 and January 2020. Geneva: World Health Organization; 2013.

[36] World Health Organization. WHO case definitions of HIV for surveillance and revised clinical staging and immunological classification of HIV-related disease in adults and children. Geneva: World Health Organization; 2007.

[37] Li J, Li T, Li YK, Gao WF, Su Q, Yuan FS, et al. Epidemiological characteristics and influencing factors of hospitalized patients with TB/HIV co-infection in high HIV/AIDS prevalence areas. Chin J AIDS STD. 2020;26(5):530–3.

[38] Zhang YN, Lu J, Ma Y, Li L, Liu Y, Ying ZJ, et al. Age-period-cohort analysis of pulmonary tuberculosis epidemiological trends from 2005 to 2024 and forecasts for 2035 in Sichuan Province. China Front Public Health. 2026;14:1757996. 41952827 10.3389/fpubh.2026.1757996 PMC13055541

[39] Wang RB, Liao RJ, Wang SJ, Feng XY, Sidu WJ, Are RAM, et al. Spatiotemporal characteristics of pulmonary tuberculosis in Liangshan Prefecture, 2017–2021. J Prev Med Inf. 2023;39(8):878–85.

[40] Cui ZZ, Huang F, Liang DB, Huang Y, Qin HF, Ye J, et al. Tuberculosis among ambulatory people living with HIV in Guangxi Province, China: a longitudinal study. Int J Environ Res Public Health. 2022;19(19):12280. 36231580 10.3390/ijerph191912280 PMC9565094

[41] Wang C, Shi YP, Liu Y, Zhou Y, Du J, Hu X, et al. Coinfection of hepatitis B, tuberculosis, and HIV/AIDS in Beijing from 2016 to 2023: a surveillance data analysis. BMC Infect Dis. 2016;25:584. 10.1186/s12879-025-10952-0 PMC12016106 40269753

[42] Hu YX, Wang Y, Fang SL, Lu XL. Analysis of the burden of polypharmacy and its influencing factors among elderly tuberculosis patients in Guizhou province. Chin J Pharm. 2023;34(9):1126–30.

[43] Ren JH, Jia SY, Che SS, Yu G, Wang J, Gao X, et al. Analysis of the survival conditions and influence factors of antiretroviral therapy for drug users with HIV/AIDS in Liangshan Prefecture from 2010 to 2019. Chin J Dis Control Prev. 2023;27(6):661–8.

[44] Yuan D, Liu MJ, Jia P, Li YP, Huang YL, Ye L, et al. Prevalence and determinants of virological failure, genetic diversity and drug resistance among people living with HIV in a minority area in China: a population-based study. BMC Infect Dis. 2020;20:443. 32576136 10.1186/s12879-020-05124-1 PMC7310496

[45] Yang N, Chen C, He JG, Li J, Zhong Y. Treatment outcome and its associated factors among HIV-MTB co-infected patients in Sichuan, China: a retrospective study. Medicine. 2022;101(48):e32006. 36482608 10.1097/MD.0000000000032006 PMC9726276

[46] Bastard M, Sanchez-Padilla E, Cros P, Khamraev AK, Parpieva N, Tillyashaykov M, et al. Outcomes of HIV-infected versus HIV-noninfected patients treated for drug-resistance tuberculosis: a multicenter cohort study. PLoS ONE. 2018;13(3):e0193491. 29518098 10.1371/journal.pone.0193491 PMC5843270

[47] Singh A, Prasad R, Balasubramanian V, Gupta N. Drug-resistant tuberculosis and HIV infection: current perspectives. HIV/AIDS Res Palliat Care. 2020;12:9–31. 10.2147/HIV.S193059 PMC6968813 32021483

[48] Bi RL, Dou LW, Pei R, Jike CN, Yu G, Wang J, et al. A mixed-methods study on healthcare workers’ perceptions of treatment adherence among HIV-TB co-infected patients in a multi-disease prevention policy context. Front Public Health. 2025;13:1704215. 41415228 10.3389/fpubh.2025.1704215 PMC12708514

[49] Zhuoma LC, Zeng YL, Yu G, Huang YL, Bian SY, Yu H, et al. Survival of HIV infected patients receiving antiretroviral therapy in four counties in Liangshan Prefecture. Chin J AIDS STD. 2022;28(2):133–7.

[50] Liao RJ, Tang ZH, Zhang N, Hu L, Chang ZQ, Ren JY, et al. Discrepancies between self-reported medication in adherence and indirect measurement adherence among patients undergoing antiretroviral therapy: a systematic review. Infect Dis Poverty. 2024;13(1):51. 38970140 10.1186/s40249-024-01221-4 PMC11225374

[51] Sossen B, Kubjane M, Meintjes G. Tuberculosis and HIV coinfection: progress and challenges towards reducing incidence and mortality. Int J Infect Dis. 2025;155:107876. 40064284 10.1016/j.ijid.2025.107876 PMC12957463

[52] Feng Y, Ge LY, Cheng WH, Zhuo Y, Cao SJ, Tang J, et al. Survival time and influencing factors among people living with HIV in Guilin City, Guangxi, China: a retrospective cohort study (1996–2022). Front Public Health. 2026;13:1575990. 41567789 10.3389/fpubh.2025.1575990 PMC12816266

[53] Chen YY, Liu XH, Wang YL, Hou MJ, Wang ZY, Yuan HZ, et al. Initial drug resistance of Mycobacterium tuberculosis in HIV patients with tuberculosis in Henan. Chin J AIDS STD. 2021;27(1):14–6.

